# JAK Inhibitors in Rheumatoid Arthritis: Immunomodulatory Properties and Clinical Efficacy

**DOI:** 10.3390/ijms25158327

**Published:** 2024-07-30

**Authors:** Kajetan Kiełbowski, Paulina Plewa, Aleksandra Wiktoria Bratborska, Estera Bakinowska, Andrzej Pawlik

**Affiliations:** 1 Department of Physiology, Pomeranian Medical University, 70-111 Szczecin, Poland; kajetan.kielbowski@onet.pl (K.K.); esterabakinowska@gmail.com (E.B.); 2 Institute of Biology, University of Szczecin, 71-412 Szczecin, Poland; paulina.plewa@op.pl; 3 Department of Internal Medicine, Poznan University of Medical Sciences, 60-780 Poznan, Poland; aleksandrabratborska@gmail.com

**Keywords:** rheumatoid arthritis, JAK/STAT pathway, JAK inhibitors

## Abstract

Rheumatoid arthritis (RA) is a highly prevalent autoimmune disorder. The pathogenesis of the disease is complex and involves various cellular populations, including fibroblast-like synoviocytes, macrophages, and T cells, among others. Identification of signalling pathways and molecules that actively contribute to the development of the disease is crucial to understanding the mechanisms involved in the chronic inflammatory environment present in affected joints. Recent studies have demonstrated that the Janus kinase/signal transducer and activator of transcription (JAK/STAT) pathway regulates the behaviour of immune cells and contributes to the progression of RA. Several JAK inhibitors, such as tofacitinib, baricitinib, upadacitinib, and filgocitinib, have been developed, and their efficacy and safety in patients with RA have been comprehensively investigated in a number of clinical trials. Consequently, JAK inhibitors have been approved and registered as a treatment for patients with RA. In this review, we discuss the involvement of JAK/STAT signalling in the pathogenesis of RA and summarise the potential beneficial effects of JAK inhibitors in cells implicated in the pathogenesis of the disease. Moreover, we present the most important phase 3 clinical trials that evaluated the use of these agents in patients.

## 1. Introduction

Rheumatoid arthritis (RA) is an autoimmune disease characterised by autoantibody production, chronic joint inflammation, synovial hyperplasia, and progressive cartilage and bone destruction [1]. The prevalence of RA is reported to be around 0.21–0.46% worldwide [2]. Over 70% of patients are seropositive, presenting autoantibodies such as rheumatoid factors (RFs), anti-citrullinated protein antibodies (ACPAs), and anti-carbamylated protein antibodies (anti-CarP Abs) [3]. Apart from leading to worsening disability, RA is also associated with various systemic complications and reduced life expectancy [4]. Patients diagnosed with RA experience fatigue; weight loss; anxiety and depression; renal failure; liver diseases; and haematological deviations, such as leucocytosis, thrombocytosis, and anaemia of chronic inflammation, more often than the healthy population [5,6,7]. As a result of multiple disturbances and chronic pain, patients have significantly reduced health-related quality of life (HRQoL), defined as the influence of a disease on physical, emotional, and social well-being. Another common problem in RA is sleep disturbances [8].

The pathophysiology of RA involves a complex interplay between immune responses, inflammatory processes, and metabolic abnormalities. Immune cells, such as fibroblast-like synoviocytes (FLSs), macrophages, and T cells, as well as numerous adhesion molecules and cytokines, play essential roles in the inflammatory responses, emphasising the importance of understanding these mechanisms to manage RA. The current treatment methods for RA can be categorised into four main classes: non-steroidal anti-inflammatory drugs (NSAIDs), glucocorticoids, and non-biological and biological disease-modifying anti-rheumatic drugs (DMARDs and bDMARDs, respectively) [9]. Traditional DMARDs exhibit adverse events (AEs) when used in the long term, such as a significantly increased risk of heart failure, angina, and cardiovascular mortality [10,11]. Biological drugs are commonly associated with infections that affect the upper respiratory tract most frequently [12]. Moreover, treatment with abatacept might pose a slightly higher overall malignancy risk and lead to the development of non-melanoma skin cancer [13]. Recent advancements in RA treatment have seen the emergence of targeted synthetic DMARDs, such as Janus kinase (JAK) inhibitors, which block specific enzymes involved in the inflammatory process. These medications offer an alternative for patients who do not respond adequately to traditional DMARDs or biological therapies [14]. Currently, JAK inhibitors approved for the treatment of RA are tofacitinib, baricitinib, upadacitinib, filgotinib, and peficitinib [15]. Tofacitinib was the first oral JAK inhibitor recommended for the treatment of moderate to severe RA [16]. It inhibits JAK1, JAK3, and partially JAK2. Baricitinib is a dual JAK1 and JAK2 inhibitor, while upadacitinib and filgotinib selectively inhibit JAK1 [17,18,19]. Peficitinib is a pan-JAK inhibitor, as it inhibits JAK1, JAK2, and JAK3, as well as tyrosine kinase 2 (TYK2) [20].

In this review, we present and discuss the immunomodulatory properties and clinical efficacy of JAK inhibitors in the treatment of RA. We focus on the influence of JAK inhibitors on immune cells involved in the pathogenesis of RA and summarise the available preclinical and clinical studies.

## 2. Brief Overview of the JAK/STAT Pathway

The JAK/STAT pathway constitutes a vital signalling cascade that regulates immunity, development, disease, mechanistic signalling, and cellular homeostasis [21]. It includes ligand–receptor complexes comprised of JAKs and STATs. The JAK family includes four members: JAK1, JAK2, JAK3, and TYK2. The STAT family comprises seven members: STAT1, STAT2, STAT3, STAT4, STAT5a, STAT5b, and STAT6 [22]. JAK1 regulates the activation and differentiation of immune cells and promotes the development of myeloid cells and hematopoietic stem cell differentiation. It mediates signals from numerous cytokine receptors, including those of interleukin (IL)-2, IL-4, IL-6, IL-7, IL-9, IL-11, and IL-15. Furthermore, it is phosphorylated by the interferon (IFN)-α/β receptor, the IFN-γ receptor, the ciliary neurotrophic factor (CNTF) receptor, the oncostatin M (OSM) receptor, the leukaemia inhibitory factor (LIF) receptor, and the cardiotrophin (CT)-1 receptor [23]. JAK2 is activated upon binding of IL-3, IL-5, erythropoietin (EPO), thrombopoietin (TPO), prolactin, and granulocyte-macrophage colony-stimulating factor (GM-CSF) to their receptors. JAK3 is predominantly expressed in endothelial cells and is involved in the signalling of the IL-2, IL-4, IL-7, IL-9, IL-15, and IL-21 receptors [24]. TYK2 is crucial for signalling pathways related to IFN-α/β, IL-6, IL-10, IL-12, IL-22, and IL-23 receptors [25]. STAT1 is activated by IFN-α, IFN-β, IL-2, IL-6, tumour necrosis factor (TNF), epidermal growth factor (EGF), platelet-derived growth factor (PDGFR), hepatocyte growth factor (HGF), and angiotensin II [26]. Importantly, STAT2 forms a transcription factor complex with STAT1 and interferon regulatory factor 9 (IRF9), known as IFN-stimulated gene factor 3 (ISGF3), which translocates to the nucleus and subsequently activates interferon-stimulated genes (ISGs) [27]. Phosphorylated STAT3, upon activation by IL-6, IL-10, IL-11, IL-19, IL-20, L-21, IL-22, IL-24, IL-26, IL-27, IL-31, G-CSF, and leptin, regulates genes involved in proliferation, anti-apoptosis, and immune functions. In addition, STAT3 is activated by LIF, IFN-α/β, IFN-γ, and OSM [28]. STAT4 is crucial for regulating the humoral immune response, as it activates genes responsible for proliferation and differentiation of T helper (Th) 1 cells and T helper follicular T (Tfh) cells. It is activated by IL-12 and IL-23, as well as type I IFN. STAT5 exists in two isoforms: STAT5A and STAT5B, which exhibit similar structural features and are activated by IL-2, IL-3, EPO, TPO, IFN-α, IFN-β, and GM-CSF [29,30]. STAT6 mediates signals of IL-4 and IL-13 and regulates Th2 and B cell differentiation. 

As non-receptor tyrosine kinases, JAKs become activated after cytokines bind to their cell surface receptors. This interaction triggers their transphosphorylation, which in turn induces tyrosine phosphorylation of the receptors, followed by binding of STAT proteins. Once STATs bind to the docking sites, they undergo phosphorylation and dimerisation. They form either heterodimers or homodimers and translocate to the nucleus, where they regulate cytokine-related gene transcription after binding to specific DNA sites [31]. Importantly, negative regulation of the JAK/STAT signalling pathway can be induced by protein inhibitors of activated STAT (PIAS), suppressors of cytokine signalling (SOCS), and protein tyrosine phosphatase (PTP) [32]. 

The JAK/STAT pathway regulates the effect of numerous cytokines on immune cells. STAT1, STAT3, and STAT6 promote the expansion of myeloid-derived suppressor cells (MDSCs), which play an immunosuppressive function. STAT3 and STAT5 are crucial for the proper proliferation and function of dendritic cells (DC) and natural killer (NK) cells [21]. STAT1 and STAT4 condition Th1 cell polarisation, while STAT6 is involved in the Th2 cell response. STAT3 mediates the induction of retinoic acid receptor-related orphan receptor γ (RORγt), which constitutes a specific transcription factor of Th17 cells. Similarly, STAT5 regulates the expression of forkhead box P3 (FOXP3), a specific transcription factor of regulatory T cells (Treg) [33]. 

As mentioned, numerous proinflammatory cytokines activate the JAK/STAT signalling pathway and therefore promote inflammation and joint destruction, contributing to RA development and progression. IL-6 induces the JAK/STAT signalling cascade in rheumatoid synoviocytes [34]. In patients with RA, especially seronegative patients with low activity and recent onset of symptoms, basal STAT phosphorylation is downregulated. Moreover, about half of the patients who are unresponsive to treatment present inactive JAK/STAT signalling [35]. Activation of JAK3 is crucial for the innate immune response performed by type 1 innate lymphoid cells (ILC1), which stimulate inflammation in RA [36]. IL-21 promotes differentiation of Tfh cells via JAK/STAT signalling and contributes to RA progression [37]. In a rat model, excess activity of either JAK2 or STAT3 aggravates swelling and destruction in joints and contributes to inflammation and excess collagen deposition in the lungs, inducing lung fibrosis [38]. In addition, it has been suggested that JAK2/STAT3 signalling mitigates effector T cell migration to joints, therefore promoting their inflammation via neural precursor cells that express developmentally downregulated 9 (NEDD9) [39]. In addition, the expression of the *FOXP3* and *SOCS* genes, negative regulators of the JAK/STAT cascade, is downregulated in patients with RA [40]. Moreover, these patients present higher expression of JAK1, JAK3, STAT1, and STAT3 in synovial tissues compared to healthy controls [41]. Therefore, the JAK/STAT pathway has emerged as a promising therapeutic target in the management of RA. 

## 3. Immunomodulatory Properties of JAK Inhibitors and Preclinical Efficacy

### 3.1. Fibroblast-Like Synoviocytes

FLSs are the major cellular component of hyperplastic synovial pannus, one of the key features of RA. During the pathogenesis of the disease, FLSs develop an invasive phenotype and behaviour that have been suggested to be “tumour like”. Intriguingly, mutational similarities between FLSs and cancer cells have been found [42]. Identification of the key molecules and signalling pathways that drive the invasive transformation of FLSs may allow researchers to identify new therapeutic targets that will eventually improve treatment outcomes. There is evidence that JAK/STAT signalling plays an important role in FLS invasiveness in patients with RA. Non-coding RNAs (ncRNAs) represent significant regulators of gene expression, and dysregulation of these molecules is implicated in the pathogenesis of numerous diseases. ncRNAs targeting the JAK/STAT pathway have been demonstrated to induce anti-inflammatory effects [43].

Several studies have investigated the impact of JAK inhibitors on FLS behaviour. Tofacitinib (JAK1, 2, and 3 inhibitor [44]) suppresses OSM-induced JAK/STAT signalling in FLSs from patients with RA; OSM is a pleiotropic cytokine implicated in the progression of RA [34,45]. Tofacitinib also suppresses the expression of chemokines stimulated by TNF [46], thus limiting the infiltration of immune cells, another process associated with inflammatory responses in rheumatic joints. Additionally, tofacitinib regulates other major cellular processes, such as autophagy and myofibroblast differentiation. The former mechanism involves degeneration of organelles, which is considered to protect from apoptotic stimulation. In FLSs from patients with RA, dysregulation of autophagy may be responsible for resistance to apoptosis [47]. Importantly, the beneficial effects of tofacitinib could depend on its ability to regulate autophagy. Vomero et al. [48] showed that tofacitinib reduces the level of LC3-II, which is a marker of autophagy. However, there were different results in a recent study evaluating the influence of tofacitinib on FLSs obtained from patients with psoriatic arthritis. Specifically, this JAK inhibitor could stimulate the expression of LC3-II [49]. The effects of tofacitinib could depend on the cellular context, the underlying inflammatory mechanisms, and the stage of the disease. In the latter study, the authors obtained FLSs from the most inflamed joints after the introduction of the treatment. Regarding the process of myofibroblast differentiation, an analysis of RA synovium demonstrated higher expression of α-smooth muscle actin (α-SMA) and decreased expression of E-cadherin; these changes resemble epithelial–mesenchymal transition, which could be related to fibrosis. Stimulation of FLSs in patients with RA with tofacitinib could significantly reduce collagen type I and α-SMA messenger RNA (mRNA) expression [50]. Interestingly, studies also investigated the efficacy of tofacitinib combination. Specifically, the use of tofacitinib with iguratimob, a conventional synthetic disease-modifying anti-rheumatic drug (csDMARD), demonstrated greater efficacy in reducing the expression of IL-1β as one of the markers of pyroptosis in TNF-α-stimulated FLSs from patients with RA [51]. FLSs obtained from different joints can respond differently to JAK inhibitors. Hammaker et al. [52] found different epigenetic profiles of cells obtained from knees and hips. Specifically, the authors demonstrated that cells derived from these two sites have a different histone acetylation profile. Consequently, they observed greater STAT promoter/enhancer activity in the knees. These findings could explain different responses to tofacitinib, as cells derived from the hip joint were more sensitive to this JAK inhibitor [52]. In the future, it might be possible to offer different treatment methods to patients depending on the most affected joints. 

Baricitinib, a JAK1/JAK2 inhibitor [53], also modulates the behaviour of FLSs in the context of RA. Similarly to tofacitinib, baricitinib suppresses inflammatory responses stimulated by OSM. Specifically, the drug alleviates the protein expression of IL-6 and MCP-1 in FLSs from patients with RA stimulated with OSM [54]. Moreover, the drug affects the expression of gliostatin, a proangiogenic factor that is regulated by inflammatory cytokines. Baricitinib can suppress IFN-γ-induced gliostatin mRNA expression [55]. IFNs are implicated in the pathogenesis of RA, and after binding to their receptors, downstream JAK-associated pathways are activated, which suggests that JAK inhibitors affect IFN signalling. One of the pathways induced by IFN-γ activates the tyrosine kinase focal adhesion kinase (FAK), which enhances invasion and migration. Because FAK activation depends on JAK2, the use of baricitinib could also suppress FAK phosphorylation [56]. Moreover, tofacitinib and baricitinib prevent the activity of a TNF-induced pro-inflammatory transcription factor, interferon regulatory factor 1 (IRF1), which is associated with the inflammatory effects of IFN-β [57].

**Figure 1:**
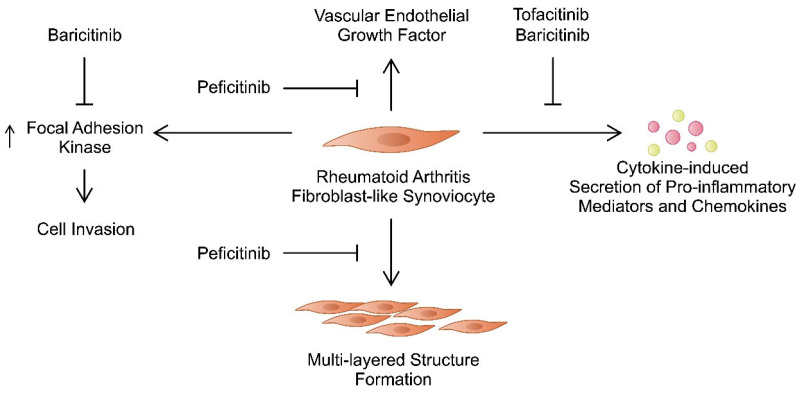
The effects of tofacitinib, baricitinib, and peficitinib on various mechanisms involving fibroblast-like synoviocytes that take part in the pathogenesis of rheumatoid arthritis.

Peficitinib is a pan-JAK inhibitor [58], whose activity towards FLSs from patients with RA has also been investigated. FLSs from patients with RA could be cultured in a three-dimensional (3D) micromass to form a multi-layered lining structure that resembled synovial lining hyperplasia. The authors examined the impact of JAK inhibitors on the structure and behaviour of these cells. Contrary to tofacitinib, peficitinib could suppress the formation of the multi-layered structure. While both agents reduced the release of matrix metalloproteinases (MMPs) and IL-6, only peficitinib suppressed the production of vascular endothelial growth factor (VEGF) [28]. Moreover, peficitinib regulates the influence of platelet-derived growth factor (PDGF)-BB on FLSs. First, the authors observed elevated PDGF-BB in the plasma of patients with RA. Second, they found that this drug enhances FLS proliferation and migration and inhibits apoptosis [59]. Moreover, PDGF-BB seems to be involved in the pathogenesis of RA, as it enhances the secretion of MMP-3, IL-6, and VEGF from FLSs from patients with RA. Importantly, peficitinib could suppress these responses. Additionally, it inhibited the tube formation by human umbilical vein endothelial cells (HUVECs), indicating that it can ameliorate angiogenesis, another process involved in the progression of RA [60,61]. Interestingly, FLSs from patients with RA pretreated with peficitinib showed reduced proliferation and macrophage-oriented chemotactic features [62], thus demonstrating multidirectional mechanisms induced by this novel JAK inhibitor. Between tofacitinib, baricitinib, and peficitinib, the latter agent demonstrated the strongest ability to suppress proliferation of RA-FLSs. Importantly, the drug does not induce cytotoxic effects in FLSs from patients with RA [63]. Furthermore, momelotinib, a JAK1/2 inhibitor, might induce beneficial effects in FLSs [64]. Overall, numerous studies have demonstrated that JAK inhibitors suppress invasive features of FLSs in patients with RA (Figure 1). Importantly, these agents induce multiple effects that show their anti-rheumatic potential. Table 1 summarises major mechanisms targeting FLSs induced by JAK inhibitors. 

### 3.2. Macrophages

Macrophages have been the subject of research for over a century. In addition, they are associated with immune control and prevention of infections [65]. Macrophages are detectable in the synovial membrane [65,66]. During RA pathogenesis, blood levels of classical CD14+CD16+ monocytes decrease, while the levels of CD16+ monocytes (non-classical and intermediate) increase [65,67]. Due to their original profile associated with receptor expression, this population predominantly affects the severity of RA [65]. In patients with RA, there is a period with a significantly reduced percentage of circulating monocytes, followed by an increase in polarised macrophages that accumulate in the joints [68]. In general, macrophages present in the synovial membrane are primarily responsible for triggering the mechanisms that enable the onset of inflammation. Specifically, cytokines and enzymes associated with osteoblast and fibroblast activation are released, leading to joint degradation and disease progression [66]. 

Two types of macrophages transform from monocytes via different pathways after migrating to the synovial membrane. The M1 phenotype is characteristic of inflammatory processes, while the M2 macrophage is primarily associated with anti-inflammatory responses. M1 macrophages contain receptors for major histocompatibility complex (MHC) II that are associated with the initiation of proliferation of CD4+ T cells, which in turn secrete large amounts of TNFα that lead to inflammation and bone damage [69]. Additionally, Toll-like receptor 4 (TLR4), a receptor that is expressed by macrophages, is associated with the pro-inflammatory properties of these cells by regulating the secretion of IL-1β, IL-6, and TNFα (66). In addition, TLR4 also allows the identification of ligands important for RA, for example, ACPA and native joint proteins [70]. Through releasing chemokines (CXCL4 and CXCL7), M1 macrophages are responsible for infiltration of monocytes and neutrophils. Furthermore, they secrete various types of MMPs and support synovitis, which is associated with degradation of joint tissue [65,71]. These cells form an ideal environment for colonisation by B cells, which then initiate the secretion of ACPA [65,72]. In contrast, M2 macrophages are primarily responsible for protective functions. They support wound healing and significantly contribute to reducing the inflammatory process. This is possible due to the release of angiogenic, chemotactic, and extracellular matrix (ECM) [66]. In addition, M2 macrophages have the endogenous ability to release IL-10 and transforming growth factor (TGF)-β, which then stimulate the immune system towards tissue repair [66].

Patients with RA have an increased level of M1 macrophages. Interestingly, it is possible to change the polarisation of macrophages from M1 to M2 [65]. One of the main pathways involved in the regulation of macrophage polarisation is the JAK/STAT signalling cascade, which plays a major role in bone destruction [73]. M1 polarisation is linked to the STAT1 pathway, specifically by IFN-γ, which interacts with receptors located on the cell surface and mediates the activation of this pathway, thereby reducing M2 polarisation [74,75]. By contrast, M2 polarisation involves the STAT3 and IL-10 pathways, which are associated with a decrease in the expression of pro-inflammatory mediators (IFN, TNFα, and IL-6), while at the same time producing anti-inflammatory factors that positively affect tissue repair [76]. Tofacitinib reduces the release of TNFα and IL-6 from macrophages, while JAK2/JAK3 inhibitors have been shown to positively affect the change in polarity of M1 to M2 macrophages [77,78]. This is somewhat inconsistent with the results obtained with ruxolitinib, as this inhibitor has been shown to decrease polarisation towards M2 macrophages by inhibiting JAK1/JAK2 [79]. On the other hand, baricitinib increases polarisation towards M2 macrophages [80]. However, a precise influence of the JAK inhibitors on the behaviour of macrophages is not well understood, particularly with regard to disturbances in the parallel stimulation of M1/M2 macrophages [79]. Therefore, more studies should be performed to examine the impact of JAK inhibitors on macrophage phenotypes. Perhaps other macrophage phenotypes play a role in RA progression and are affected by JAK inhibitors. Apart from the classic M1 and M2 phenotypes, other macrophage variants are detected, as demonstrated in atherosclerosis [81]. 

### 3.3. T and B Cells

Several populations of T cells are involved in the progression of RA, but their exact function is not fully understood. Nevertheless, there is evidence that CD4+ T cells are crucial in driving the progression of RA. ILs activate CD4+ T cells, which then transform into specific effector cells [82]. T helper 1 (Th1) cells produce large amounts of pro-inflammatory cytokines such as IFN-γ, TNFα, and IL-2, which promote RA [83]. Th17 cells release IL-17, which affects the production of pro-inflammatory cytokines such as IL-1β, IL-6, and TNFα in synovial cells, bone cells, cartilage, and macrophages. In addition, IL-17 affects the secretion of the chemokines chemokine (C-X-C motif) ligand 1 (CXCL1), CXCL2, and chemokine (C-C motif) ligand 2 (CCL2), which are associated with increased inflammation mediated by neutrophils and macrophages that infiltrate the synovial membrane [82,83,84]. Th2 cells are responsible for the production of IL-4 and IL-5 and influence the stimulation of B cells to produce immunoglobulin type E (IgE), which increases the pathogenesis of RA [84]. On the other hand, CD8+ T cells release large amounts of IFN, allowing long-term maintenance of inflammation. This is possible due to mobilisation to release significant amounts of pro-inflammatory cytokines [85]. Patients with RA have increased levels of IL-4 CD8+ T cells, which are associated with TNFα production [86]. JAK/STAT signalling influences T cells. For example, the JAK/STAT3 signalling pathway is associated with IL-6, which supports the polarisation of naive T cells to Th cells [87]. Additionally, IL-21 has a positive effect on the JAK/STAT3 signalling pathway [88]. On the other hand, the JAK/STAT1 and IFN-γ signalling pathways facilitate the Th1 cell response and Th17 cell differentiation [89]. IL-4 stimulation of JAK/STAT6 affects the polarisation of naive T cells into Th2 cells [90]. 

Importantly, the use of tofacitinib results in inhibition of the expression of transcription factors that affect CD4+ differentiation. Additionally, it restricts the release of IL-2, IL-4 IL-17, IL-21 and IFNγ [91,92]. Therefore, it affects the presence of several cytokines involved in the pathogenesis of RA. Importantly, it affects the levels of IL-17, an important cytokine, which is upregulated in patients with RA [93]. However, the relationship between tofacitinib and Th17 cells and IL-17 seems complex. An in vitro experiment showed that in the presence of TGF-β1, tofacitinib enhanced the production of IL-17A. Conversely, this result was not observed in the absence of TGF-β1 [94]. Clinically, the 24-week treatment of RA patients was associated with the gradual decrease in serum IL-17A [95]. Therefore, the effect of tofacitinib may depend on cellular context. Moreover, the influence of JAK inhibitors on Th17 cells depends on the dose of the drug [96]. Perhaps tofacitinib affects other IL-17-secreting cells to change the profile of circulating cytokines. Both Th17 cells and γδ T-helper 17 (γδT17) are important sources of this cytokine. Yang and colleagues showed that tofacitinib can reduce the activity of γδT17 cells through the nucleotide-binding domain (NOD)-like receptor protein 3 (NLRP3) inflammasome [97]. Intriguingly, it demonstrates that the JAK inhibitor affects the activation of NLRP, a pattern recognition receptor (PRR) that is a major pro-inflammatory mediator [98]. In a different study published by Furuya and collaborators, the authors also confirmed the ability of tofacitinib to suppress NLRP3 in stimulated neutrophils [99]. Expression of NLRP3 is elevated in synovial tissues of RA patients and has been suggested to enhance the progression of RA [100,101]; thus, suppression of NLRP3 by JAK inhibitors seems to induce beneficial effects. Another mechanism related to tofacitinib, which has been recently published, refers to immunosenescence of T cells [102]. Filgotinib, which is responsible for the inhibition of polarity to Th1/Th2 lymphocytes and, on a small scale, Th17 [77]. Ruxolitinib inhibits JAK by decreasing plasma IL-6 levels, which greatly affects the activation of CD4+ T cells and, consequently, differentiation into effector cells [77]. Relatively recently, baricitinib has been approved for the treatment of RA by the FDA. It also contributes, like the aforementioned inhibitors, to disruption of the IL-6 signalling pathway. Although in the initial phase of treatment there is an incompletely understood increase in the number of T cells CD+ and CD8+, after about 12 weeks it returns to baseline values [103]. A study by Tanak et al. showed that there is a significant decrease in Th1 cells, which are dependent on cytokines effectively inhibited by baricitinib. There is also an increase in Treg cells, which favourably affect the RA microenvironment [104]. In addition, JAK inhibitors affect the expression of neural precursor cell-expressed, developmentally downregulated 9 (NEDD9), a molecule implicated in migration and costimulation of T cells, which has been demonstrated to be involved in the pathogenesis of an animal model of RA [105]. Regarding the influence of JAK inhibitors, reduced expression of NEDD9 in CD4+ cells was observed in patients treated with baricitinib and upadacitinib. CD4+ cells obtained from patients treated with baricitinib showed reduced migratory properties [39]. Interestingly, apart from affecting T cells themselves, JAK inhibitors influence interactions between T cells and other cells, such as macrophages. Recently, tofacitinib and ruxolitinib were shown to suppress the maturation of cytokine-activated T cells. Consequently, these cells show reduced potential for activating macrophages [106]. 

**Figure 2:**
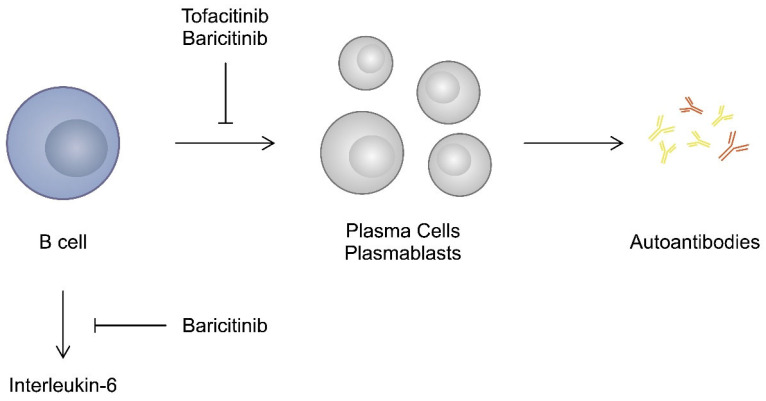
An impact of JAK inhibitors on the behaviour of B cells.

B cells represent another lymphocyte population that contributes to the progression of RA. Firstly; by acting as antigen presenting cells (APC), they can enhance the activity of T cells. Secondly, higher levels of pro-inflammatory cytokines and chemokines are detected in ACPA-positive patients. Therefore, it is suggested that they could be secreted by B cells. Moreover, autoreactive B cells transferred into plasma cells produce autoantibodies, which also contribute to the progression of RA [107]. A higher number of activated B cells is observed in the peripheral blood of patients with RA than in controls [108]. Therefore, modulating the activity of B cells might also be associated with beneficial effects in the context of RA. Importantly, STATs are implicated in the ability of B cells to differentiate into plasma cells; thus, they affect their humoral immune responses [109]. Accordingly, studies have demonstrated that JAK inhibitors affect the behaviour of B cells. Stimulation of B cells with tofacitinib reduced their potential to differentiate into plasmocytes. However, elimination of the drug reversed this inhibition [110]. The ability of tofacitinib to suppress antibody responses in B cells has been suggested to induce immunosuppressive reactions in transplantation [111]. Baricitinib was also suggested to influence B cell responses. Tanaka et al. analysed data from three clinical trials that investigated the use of baricitinib. The authors found that treatment with JAK inhibitors was associated with an increased presence of B cells. Conversely, titres of RF significantly decreased, while titres of ACPA decreased in patients treated with baricitinib 4 mg [104]. This study confirms the suppression of autoantibody production, while the increase in B cell frequency could potentially result from compensation. Kubo and colleagues also showed that baricitinib suppresses the differentiation of B cells into plasmablasts. In addition, the authors observed that baricitinib inhibited IL-6 production from B cells [112] (Figure 2). 

### 3.4. Animal Studies

Several animal RA models are being used in investigations. One of the most commonly used is collagen-induced arthritis (CIA), which exhibits proportional synovitis and degradation of both cartilage and bone [113]. To induce RA, a strain of mice that is genetically susceptible is immunised with collagen type II (CII) in a complex with a Freund adjuvant [114,115,116]. Another method is the use of collagen antibody-induced arthritis (CAIA). This process involves transferring monoclonal antibody-containing serum from a vaccinated mouse to a susceptible unvaccinated mouse [116]. The course of arthritis can be compared to that obtained in RA and CIA—synovitis, degradation of cartilage, and bone. However, CAIA is not a response from T and B cells but is largely associated with the accumulation of multi-nucleus inflammatory cells and macrophages [116]. It is also possible to induce proteoglycan-induced arthritis (PGIA), which subsequently causes polyarticular inflammation along with synovitis caused by the accumulation of immune cells [117]. Other methods are adjuvant-induced arthritis (AIA), which is characterised by quite high reproducibility, simplicity of execution, and short duration, and K/BxN serum-transfer arthritis (STA) [117,118]. 

Numerous studies have been conducted to evaluate the efficacy of JAK inhibitors in animal models of RA. Hablot et al. [119] evaluated the effect of tofacitinib on a DBA1/J mouse model of CIA. Th therapy significantly reduced the number of Th17 cells and, thus, IL-17 and other pro-inflammatory cytokines. In a study using a mouse with AIA, tofacitinib reduced inflammation, swelling, bone resorption, and IL-6 and RANKL levels in T cells [120]. In a rat model of AIA, Gertel et al. [121] revealed a significant reduction in the incidence of CD4+ T cells, IFN, and IL-1β. Makabe et al. [122] studied the effect of baricitinib on rats with CAIA; it significantly inhibited the IL-6/JAK-STAT pathway. Finally, in rats with CIA, decernotinib reduced JAK3 activity [123]. 

## 4. Clinical Studies

### Tofacitinib 

In the previous sections of this article, we discussed the immunomodulatory properties and preclinical studies demonstrating the efficacy of JAK inhibitors in RA. Importantly, a number of clinical trials also investigated the use of these agents in patients with RA, which has led to FDA approval and treatment registration. 

The safety and efficacy of tofacitinib have been investigated in the ORAL clinical trials. In 2012, the results of the large phase 3 ORAL Solo trial that evaluated and confirmed the role of tofacitinib in patients with RA were published [92]. The authors explored the efficacy and safety of tofacitinib monotherapy. The study design involved four cohorts: (1) tofacitinib 5 mg, (2) tofacitinib 10 mg, (3) placebo followed by a switch to tofacitinib 5 mg, and (4) placebo followed by a switch to tofacitinib 10 mg. After 3 months, the investigators compared the American College of Rheumatology 20 Criteria (ACR20) score (there is a positive outcome with a ≥ 20% improvement). At 3 months, the highest response occurred in the cohort treated with tofacitinib 10 mg (65.7%) and the lowest in the groups receiving placebo (26.7%). However, when comparing the percentage of patients who achieved a Disease Activity Score-28 (DAS28) score of <2.6, there were no significant differences after 3 months of treatment. Regarding AEs, there were similar results between the groups [92]. In the ORAL Standard trial, tofacitinib was compared with adalimumab in patients with RA who concomitantly received methotrexate (MTX). After 6 months of treatment, a greater percentage of patients in the tofacitinib 5 or 10 mg and adalimumab cohorts achieved the ACR20 response compared with placebo (51.5%, 52.6%, and 47.2% vs. 28.3%) [124]. These early trials confirmed that tofacitinib indeed provides clinical benefits. 

Subsequently, researchers investigated whether tofacitinib is superior to long-used RA drugs or if a combinational therapy would be safe and effective. The ORAL Start clinical trial compared tofacitinib with MTX in patients with active RA [125]. Clinically, after 6 months of treatment, significantly more patients achieved the ACR70 response in the tofacitinib groups (25.5% and 37.7%) than in the MTX group (12%). The occurrence of AEs was similar between the groups, but in patients treated with tofacitinib, the authors noticed a more frequent occurrence of herpes zoster infections (4% vs. 1.1%). Moreover, there were five cases of cancer development in the tofacitinib groups, compared with only one in the MTX group. In the ORAL Scan trial, the combination of tofacitinib and MTX was evaluated in patients with active RA who had an inadequate response to MTX. At 6 months, the ACR20 response rate was significantly higher in the cohorts receiving tofacitinib (51.5% and 61.8% vs. 25.3%). Importantly, the study groups also showed a higher frequency of patients that achieved DAS28-ESR ≤ 3.2, which indicates low disease activity [126]. The efficacy of this strategy was confirmed in the final article summarising the 24-month study. Improvements in clinical parameters first observed after 6 months of treatment were sustained. Interestingly, the clinical results noted at 24 months were similar between patients who started treatment with tofacitinib and those who switched from placebo to tofacitinib [127]. The use of tofacitinib was examined in a population of patients with an inadequate response to a TNF inhibitor. The ORAL Step trial demonstrated that the use of tofacitinib with background MTX led to a significantly higher ACR20 response at 3 months [128]. Hence, tofacitinib provided clinical benefits in patients with insufficient responses to MTX and TNF inhibitors. Subsequently, the ORAL Strategy trial compared tofacitinib monotherapy with combinations of tofacitinib with MTX and adalimumab with MTX [129]. The combination of tofacitinib with MTX was non-inferior to the combination of MTX and adalimumab. However, non-inferiority was not achieved regarding tofacitinib monotherapy. Thus, the authors concluded that an inadequate response to MTX indicates that the addition of adalimumab or tofacitinib provides similar benefits and that these strategies are associated with better outcomes than switching to tofacitinib. Apart from the trial that investigated the use of tofacitinib for 24 months, long-term safety and efficacy data were still missing. Those were summarised in the ORAL Sequel study (NCT00413699), which included patients receiving tofacitinib (monotherapy or combinations) in previous phase 1, 2, and 3 trials [130]. Regarding the efficacy, data for tofacitinib 5 and 10 mg were available at 96 and 72 months, respectively. The ACR20 response rate was 78.5% and 82.6%, respectively. Therefore, the use of tofacitinib was associated with an increased ACR20 score after the first month of treatment and a sustained response over the next few years. Similarly, the DAS28-ESR scores decreased after the first months and remained stable for the remaining duration of the study. Regarding the safety data, 90.4% and 90.0% of patients treated with tofacitinib 5 and 10 mg, respectively, experienced AEs. However, discontinuation of treatment only occurred in 28% and 24% of patients, respectively [130].

Recently, the results of the eillance phase 3b-4 trial (NCT02092467), which investigated the safety profile of tofacitinib, were published [131]. The study involved three arms with patients treated with tofacitinib 5 mg (n = 1455), tofacitinib 10 mg (n = 1456), or a TNF inhibitor (n = 1451). Major adverse cardiovascular events (MACE) occurred in 3.4% of all patients treated with tofacitinib versus 2.5% of patients treated with a TNF inhibitor. Importantly, non-inferiority was not demonstrated. Similarly, more patients treated with tofacitinib developed cancer (4.2% vs. 2.9%). Importantly, the study included patients over 50 years of age with cardiovascular risk factors [131]. However, according to the recently published analysis of the ORAL Surveillance cohort, after an extension of the primary outcomes to involve all ischemic cardiovascular events, together with hospitalisation due to heart failure, there was no difference between tofacitinib and a TNF inhibitor. Nevertheless, the addition of venous thromboembolism was associated with a significantly elevated risk in patients treated with tofacitinib 10 mg twice a day [132]. Populations of patients with a history of smoking and older than 65 years were associated with a significantly elevated risk of MACE and malignancies [133]. On the contrary, according to the STAR-RA study, a large clinical investigation of tofacitinib-associated cardiovascular outcomes in real-world settings, there was no increased risk of cardiovascular outcomes. Nevertheless, there was an elevated risk among patients with cardiovascular risk factors [134]. In a recent publication, Balanescu et al. [135] evaluated the risk of infection associated with tofacitinib versus TNF inhibitors in cohorts from the ORAL Surveillance trial. The authors demonstrated that the use of tofacitinib is associated with greater incidence rates of infections, among which the most frequently observed included infections of the upper respiratory tract, bronchitis, and urinary tract. 

Overall, tofacitinib is the most widely investigated JAK inhibitor in RA. Throughout the years, the drug has demonstrated activity in various configurations and, importantly, in patients with an inadequate response to previous lines of therapy. However, the risk of AEs has raised concerns regarding the use of tofacitinib, and it is necessary to evaluate whether the response is sustained after treatment discontinuation. The XANADU study investigated the response after tofacitinib or MTX discontinuation at 52 weeks of treatment in patients with RA. Fewer patients sustained remission after cessation of tofacitinib at 104 weeks compared with MTX (29.2% vs. 50%). Nevertheless, this difference was not statistically significant. Perhaps the previous treatment history influenced the results. Specifically, the authors observed that among patients who did not receive previous treatment with biologics, clinical remission occurred in 57.1% of patients who discontinued tofacitinib. Re-introduction of tofacitinib was associated with clinical remission in 71.4% of patients with flare-ups [136]. Therefore, the study highlights the need to search for potential markers that could help in deciding whether tofacitinib discontinuation would be associated with a long-term response. Consequently, the risk of developing tofacitinib-related AEs could be reduced in selected populations.

Baricitinib, a JAK1/2 inhibitor, has been examined in several phase 3 clinical trials, including in patients with RA. The RA-BEACON study (NCT01721044) investigated baricitinib in patients with an inadequate response to biologic treatment. The results showed that baricitinib 4 mg significantly improved the ACR20 response, DAS28-CRP, and the Health Assessment Questionnaire Disability Index (HAQ-DI) scores at 12 weeks. More patients in the study group developed infections. Furthermore, there were significantly increased cholesterol levels in patients treated with baricitinib [137]. The RA-BEAM trial (NCT01710358) compared the efficacy and safety of baricitinib and adalimumab in a cohort with an inadequate MTX response. The placebo, baricitinib, and adalimumab arms comprised 488, 487, and 330 patients, respectively. At week 12, the baricitinib arm demonstrated a significantly elevated ACR20 response compared with the placebo arm and non-inferiority compared with the adalimumab arm. Moreover, baricitinib was superior to adalimumab regarding the DAS28-CRP score [138]. The RA-BEGIN (NCT01711359) proved that baricitinib monotherapy or in combination with MTX is superior to MTX monotherapy in patients who were not previously treated with csDMARDs or bDMARDs, which represents important findings for patients experiencing intolerance to MTX [139]. Importantly, the BARE BONE trial proved that baricitinib can improve bone properties. One year of treatment could improve bone mineral density, bone stiffness, and failure load [140].

Regarding the cardiovascular safety of baricitinib, Taylor et al. [141] analysed safety data from nine clinical trials. The authors found that treatment with baricitinib was not associated with the occurrence of cardiovascular outcomes and that the incidence rates were comparable with the cohort that received placebo. In another study that summarised nine clinical trials evaluating the use of baricitinib, the most common treatment-emergent AEs were infections. However, the frequency of malignancies, MACE, and DVTs and the incidence of death were stable up to approximately 9 years of exposure [142]. Intriguingly, the drug also affects the development of interstitial lung disease (ILD), a frequent lung manifestation in patients with RA. In in vivo experiments, baricitinib reduced the thickness of alveolar walls and collagen deposition, thus suppressing pulmonary fibrosis [143]. 

Similarly to other targeted treatment methods, identification of potential responders and non-responders based on easily available biomarkers might help in selecting the right treatment strategy. Consequently, potential non-responders might be less exposed to toxicity and receive an appropriate treatment more rapidly. Tucci et al. [144] investigated potential immunological markers that could help in identifying responders to baricitinib in the RA population. The authors demonstrated that monitoring STAT1 phosphorylation in monocytes could differentiate responders among patients treated with baricitinib. 

Upadacitinib is a selective JAK1 inhibitor whose efficacy and safety have been investigated in patients with RA in a number of SELECT phase 3 trials. In the SELECT-MONOTHERAPY trial, which included patients with an inadequate response to MTX, the investigators compared upadacitinib monotherapy to continued MTX. Treatment with upadacitinib was associated with a significantly higher number of patients achieving the ACR20 response, as well as improvements in the DAS28-CRP score at 14 weeks. Importantly, upadacitinib demonstrated a manageable safety profile [145]. In the SELECT-EARLY trial, the researchers investigated upadacitinib in patients who did not receive prior treatment with MTX. Upadacitinib induced significantly higher radiographic and clinical scores of RA in the MTX-naïve population, thus raising the question as to whether upadacitinib could replace MTX as the first-line treatment [146]. By combining the results of the SELECT-MONOTHERAPY and SELECT-EARLY trials, Strand et al. [147] demonstrated that treatment with upadacitinib monotherapy significantly improved patient-reported outcomes, such as pain and morning stiffness, as well as other scores. Furthermore, the JAK inhibitor showed superior clinical efficacy (ACR20 and DAS28-CRP) to adalimumab in the SELECT-COMPARE study [148]. Additionally, upadacitinib significantly suppressed structural joint damage after 48 weeks based on the EARLY and COMPARE trials [149]. The SELECT-BEYOND trial demonstrated that upadacitinib is clinically beneficial in patients refractory to bDMARDs [150]. In addition, the SELECT-CHOICE trial showed that upadacitinib provided significantly greater clinical benefits in patients treated previously with bDMARDs compared to abatacept. However, the drug was associated with a higher frequency of AEs (serious AEs: 3.3% vs. 1.6%; AEs requiring drug discontinuation: 4.6% vs. 2.9; hepatic disorder: 7.6% vs. 1.6%) [151]. 

Filgotinib is another JAK1 inhibitor that has been examined clinically. According to the phase 3 FINCH 1 trial, the use of filgotinib in patients with an inadequate response to MTX was associated with a significantly improved ACR20 response and DAS28-CRP and HAQ-DI scores at 12 weeks compared with the cohort receiving placebo. Similarly, filgotinib could suppress radiographic joint damage progression [152]. In the phase 3 FINCH2 trial, the use of filgotinib was investigated in a cohort of patients with a treatment background of csDMARDs and refractory to bDMARDs. Compared with placebo, filgotinib significantly enhanced the ACR20 response at 12 weeks. Intriguingly, in a subgroup analysis, filgotinib demonstrated efficacy in patients who were previously treated with ≥3 bDMARDs (ACR20 placebo, 17.6%; filgotinib 100 mg, 58.8%; filgotinib 200 mg, 70.3%) [153]. The FINCH3 trial evaluated the use of filgotinib as monotherapy or in combination with MTX in patients without or with limited prior exposure to MTX. The filgotinib and MTX combination significantly improved the ACR20 response compared with MTX (ACR20: 81% for filgotinib 200 mg + MTX, 80% for filgotinib 100 mg + MTX, and 71% for MTX). By contrast, the use of filgotinib in monotherapy was not superior to MTX. Therefore, the study proved that the combination of filgotinib with MTX could improve clinical outcomes in patients with limited prior exposure to MTX [154]. Table 2 summarises select major phase 3 clinical trials that have evaluated the efficacy and safety of JAK inhibitors in patients with RA.

## 5. Conclusions 

Over the years, the involvement of the JAK/STAT pathway has been extensively studied in the context of RA. This signalling pathway mediates the behaviour of immune cells that actively participate in the progression of RA, as well as FLSs, a major population that interacts with immune cells and takes part in the formation of the synovial pannus. A number of JAK inhibitors have been developed, and their efficacy has been examined in vitro and in vivo. Importantly, the use of JAK inhibitors has been examined in numerous clinical trials, which led to the approval and registration of these drugs in clinical practice. These clinical trials have demonstrated that JAK inhibitors exert clinical activity in various treatment configurations and at several treatment lines. Future studies should focus on identifying biomarkers that may predict the response to these drugs. This knowledge may improve decision making regarding the next line of therapy and reduce unnecessary exposure to these agents, which could induce serious AEs. Additionally, more JAK inhibitors should be developed and investigated in the context of RA. 

## Figures and Tables

**Table 1:** Summary of the effects of the Janus kinase (JAK) inhibitors tofacitinib, baricitinib, and peficitinib on fibroblast-like synoviocytes (FLSs).

JAK Inhibitor	Targets	Impact on Fibroblast-like Synoviocytes	References
Tofacitinib	JAK1, JAK2, JAK3	Tofacitinib suppresses the activity of JAK/STAT pathway induced by oncostatin M, decreases the expression of chemokines, regulates apoptosis and myofibroblast differentiation of FLSs.	[34,44,46,48,50]
Baricitinib	JAK1, JAK2	Baricitinib inhibits inflammatory responses stimulated by OSM, together with IFNγ-induced expression of gliostatin/thymidine phosphorylase. Baricitinib negatively regulates IFN signalling I FLSs.	[53,54,55,56]
Peficitinib	Pan-JAK inhibitor	Peficitinib can suppress the formation of multi-layered structures by RA-FLSs, as well as the production of MMPs, IL-6, and VEGF from these structures. The drug inhibits the secretion of MMP-3, IL-6, and VEGF from PDGF-BB-stimulated FLSs. Moreover, it suppresses FLS proliferation and chemotactic properties.	[28,58,60,62]

**Table 2:** Summary of selected phase 3 clinical trials investigating efficacy and safety of JAK inhibitors in RA patients.

Clinical Trial	Arms	Number of Participants	Efficacy	Toxicities	Reference
ORAL Solo (NCT00814307)	Placebo	122	ACR20: 26.7%	Serious AEs at 3 months: 4.9%	[92]
	Tofacitinib 5 mg	243	ACR20: 59.8%	Serious AEs at 3 months: 0.4%	
	Tofacitinib 10 mg	245	ACR20: 65.7%	Serious AEs at 3 months: 2%	
ORAL Standard (NCT00853385)	Placebo followed by tofacitinib 5 mg	56	ACR20: 28.3%	Serious AEs at 6 months: 0%	[124]
	Placebo followed by tofacitinib 10 mg	52		Serious AEs at 6 months: 0%	
	Tofacitinib 5 mg	204	ACR20: 51.5%	Serious AEs at 3 months: 5.9%	
	Tofacitinib 10 mg	201	ACR20: 52.6%	Serious AEs at 3 months: 5%	
	Adalimumab 40 mg	204	ACR20: 47.2%	Serious AEs at 3 months: 2.5%	
ORAL Start (NCT01039688)	Tofacitinib 5 mg	373	ACR70: 25.5 ± 2.3%	Serious AEs at 24 months: 10.7%	[125]
	Tofacitinib 10 mg	397	ACR70: 37.7 ± 2.4%	Serious AEs at 24 months: 10.8%	
	Methotrexate	186	ACR70: 12.0 ± 2.4%	Serious AEs at 24 months: 11.8%	
ORAL Scan (NCT00847613)	Tofacitinib 5 mg + methotrexate	321	ACR20: 51.5%	Serious AEs at 6 months: 5.3%	[126]
	Tofacitinib 10 mg + MTX	316	ACR20: 61.8%	Serious AEs at 6 months: 2.2%	
	Placebo to tofacitinib 5 mg + MTX	81	ACR20: 25.3%	Serious AEs at 6 months: 2.4%	
	Placebo to tofacitinib 10 mg + MTX	79		Serious AEs at 6 months: 2.7%	
ORAL Step (NCT00960440)	Placebo + MTX	132	ACR20 at 3 months: 41.7%	-	[128]
	Tofacitinib 5 mg + MTX	133	ACR20 at 3 months: 48.1%	Serious AEs at 6 months: 3.8%	
	Tofacitinib 10 mg + MTX	134	ACR20 at 3 months: 24.4%	Serious AEs at 6 months: 4.5%	
ORAL Strategy (NCT02187055)	Tofacitinib 5 mg	384	ACR50 at 6 months: 38%	Serious AEs at 6 months: 9%	[129]
	Tofacitinib 5 mg + MTX	376	ACR50 at 6 months: 46%	Serious AEs at 6 months: 7%	
	Adalimumab 40 mg + MTX	386	ACR50 at 6 months: 44%	Serious AEs at 6 months: 6%	
RA-BEACON (NCT01721044)	Placebo	176	ACR20 at 12 weeks: 27%	Serious AEs at 24 weeks: 7%	[137]
	Baricitinib 2 mg	174	-	Serious AEs at 24 weeks: 4%	
	Baricitinib 4 mg	177	ACR20 at 12 weeks: 55%	Serious AEs at 24 weeks: 10%	
RA-BEAM (NCT01710358)	Placebo	488	ACR20 at 12 weeks: 40%	Serious AEs at 24 weeks: 5%	[138]
	Baricitinib	487	ACR20 at 12 weeks: 70%	Serious AEs at 24 weeks: 5%	
	Adalimumab	330	ACR20 at 12 weeks: 61%	Serious AEs at 24 weeks: 2%	
RA-BEGIN (NCT01711359)	MTX	210	ACR20 at 24 weeks: 62%	Serious AEs at 52 weeks: 10%	[139]
	Baricitinib	159	ACR20 at 24 weeks: 77%	Serious AEs at 52 weeks: 8%	
	MTX + baricitinib	215	ACR20 at 24 weeks: 78%	Serious AEs at 52 weeks: 8%	
SELECT- MONOTHERAPY (NCT02706951)	MTX	216	ACR20 at 14 weeks: 41%	Serious AEs at 14 weeks: 3%	[145]
	Upadacitinib 15 mg	217	ACR20 at 14 weeks: 68%	Serious AEs at 14 weeks: 4%	
	Upadacitinib 30 mg	215	ACR20 at 14 weeks: 71%	Serious AEs at 14 weeks: 3%	
SELECT-EARLY (NCT02706873)	MTX	314	ACR50 at 12 weeks: 28%	Serious AEs at 24 weeks: 4.1%	[146]
	Upadacitinib 15 mg	317	ACR50 at 12 weeks: 52%	Serious AEs at 24 weeks: 4.7%	
	Upadacitinib 30 mg	314	ACR50 at 12 weeks: 56%	Serious AEs at 24 weeks: 6.4%	
SELECT- COMPARE (NCT02629159)	Placebo + MTX	651	ACR20 at 12 weeks: 36%	Serious AEs at 26 weeks: 2.9%	[148]
	Upadacitinib 15 mg + MTX	651	ACR20 at 12 weeks: 71%	Serious AEs at 26 weeks: 3.7%	
	Adalimumab + MTX	327	ACR20 at 12 weeks: 63%	Serious AEs at 26 weeks: 4.3%	
SELECT-BEYOND (NCT02706847)	Placebo	169	ACR20 at 12 weeks: 28%	Serious AEs at 12 weeks: 0%	[150]
	Upadacitinib 15 mg	164	ACR20 at 12 weeks: 65%	Serious AEs at 12 weeks: 5%	
	Upadacitinib 30 mg	165	ACR20 at 12 weeks: 56%	Serious AEs at 12 weeks: 7%	
SELECT-CHOICE (NCT03086343)	Upadacitinib	303	DAS28-CRP at 12 weeks: −2.52 DAS28-CRP < 2.6: 30%	Serious AEs at 24 weeks: 3.3%	[151]
	Abatacept	309	DAS28-CRP at 12 weeks: −2.00 DAS28-CRP < 2.6: 13.3%	Serious AEs at 24 weeks: 1.6%	
FINCH 1 (NCT02889796)	Placebo + MTX	475	ACR20 at 12 weeks: 49.9%	Serious AEs at 24 weeks: 4.2%	[152]
	Adalimumab + MTX	325	ACR20 at 12 weeks: 70.5%	Serious AEs at 24 weeks: 4.3%	
	Filgotinib 100 mg + MTX	480	ACR20 at 12 weeks: 69.8%	Serious AEs at 24 weeks: 5%	
	Filgotinib 200 mg + MTX	475	ACR20 at 12 weeks: 76.6%	Serious AEs at 24 weeks: 4.4%	
FINCH 2 (NCT02873936)	Placebo	148	ACR20 at 12 weeks: 31.1% DAS28-CRP < 3.2: 15.5%	Serious AEs at 24 weeks: 3.4%	[153]
	Filgotinib 100 mg	153	ACR20 at 12 weeks: 57.5% DAS28-CRP < 3.2: 37.3%	Serious AEs at 24 weeks: 5.2%	
	Filgotinib 200 mg	147	ACR20 at 12 weeks: 66% DAS28-CRP < 3.2: 40.8%	Serious AEs at 24 weeks: 4.1%	
FINCH 3 (NCT02886728)	MTX	416	ACR20 at 24 weeks: 71%	Serious AEs up to 52 weeks: 7%	[154]
	Filgotinib 200 mg	210	ACR20 at 24 weeks: 78.1%	Serious AEs up to 52 weeks: 8%	
	Filgotinib 100 mg + MTX	207	ACR20 at 24 weeks: 80%	Serious AEs up to 52 weeks: 6%	
	Filgotinib 200 mg + MTX	416	ACR20 at 24 weeks: 81%	Serious AEs up to 52 weeks: 6%	

## Data Availability

Not applicable.
